# Insights into Transcriptional Repression of the Homologous Toxin-Antitoxin Cassettes *yefM-yoeB* and *axe-txe*

**DOI:** 10.3390/ijms21239062

**Published:** 2020-11-28

**Authors:** Barbara Kędzierska, Katarzyna Potrykus, Agnieszka Szalewska-Pałasz, Beata Wodzikowska

**Affiliations:** Department of Bacterial Molecular Genetics, Faculty of Biology, University of Gdańsk, Wita Stwosza 59, 80-308 Gdańsk, Poland; katarzyna.potrykus@ug.edu.pl (K.P.); agnieszka.szalewska-palasz@ug.edu.pl (A.S.-P.); wodzikowska.beata@gmail.com (B.W.)

**Keywords:** transcription repression, homologous toxin-antitoxin, YefM-YoeB, Axe-Txe

## Abstract

Transcriptional repression is a mechanism which enables effective gene expression switch off. The activity of most of type II toxin-antitoxin (TA) cassettes is controlled in this way. These cassettes undergo negative autoregulation by the TA protein complex which binds to the promoter/operator sequence and blocks transcription initiation of the TA operon. Precise and tight control of this process is vital to avoid uncontrolled expression of the toxin component. Here, we employed a series of in vivo and in vitro experiments to establish the molecular basis for previously observed differences in transcriptional activity and repression levels of the *p_yy_* and *p_at_* promoters which control expression of two homologous TA systems, YefM-YoeB and Axe-Txe, respectively. Transcriptional fusions of promoters with a *lux* reporter, together with in vitro transcription, EMSA and footprinting assays revealed that: (1) the different sequence composition of the −35 promoter element is responsible for substantial divergence in strengths of the promoters; (2) variations in repression result from the TA repressor complex acting at different steps in the transcription initiation process; (3) transcription from an additional promoter upstream of *p_at_* also contributes to the observed inefficient repression of *axe-txe* module. This study provides evidence that even closely related TA cassettes with high sequence similarity in the promoter/operator region may employ diverse mechanisms for transcriptional regulation of their genes.

## 1. Introduction

Gene transcription is a multistep process carried out by DNA-dependent RNA polymerase (RNAP). Bacterial RNAP is composed of β, β′, ω and two α subunits which form a core enzyme capable of nonspecific DNA binding. However, specific promoter DNA sequence recognition enabling efficient transcription is performed by the RNAP holoenzyme which is assembled from the core enzyme and one of several σ factors. The *Escherichia coli* σ^70^ subunit, responsible for transcription of most genes required for cell growth and maintenance (housekeeping genes), is composed of four separately folded domains connected by flexible linkers. Each of these domains interacts with the core enzyme and also makes specific contacts with corresponding promoter elements [1,2,3,4].

A typical bacterial promoter recognized by sigma^70^ contains a number of characteristic sequence elements. The −10 and −35 boxes, which are named to indicate their approximate distance from the transcription start site (+1) are known to make direct contacts with surface exposed regions of the σ subunit. The −10 and −35 hexamers have 5′-TATAAT-3′ and 5′-TTGACA-3′ consensus sequences, respectively [5,6].

In prokaryotes, transcription initiation is a multistep process [7,8,9,10,11,12]. It begins with recruitment of the RNAP holoenzyme to the promoter region and formation of a loose, unstable, initial closed complex (RPc). Subsequently, RPc undergoes multiple rearrangements characterized by changes in the conformation of both the RNAP and the DNA, that involve several kinetic steps and intermediates, and which transform RPc into a tighter, advanced closed complex. Next, a transition to the open promoter complex (RPo) occurs where the transcription bubble is formed. RNAP can subsequently form the initiation complex (RPi) and start to transcribe the DNA [10,11,12].

Critical contacts mediated between the promoter DNA elements and the RNAP holoenzyme modulate the frequency and efficiency of transcription initiation, and thereby regulate gene expression [7,8,11]. It has been shown that the more similar the −10 and −35 promoter regions are to the consensus sequences, the stronger the promoter [5,13]. Moreover, active promoters are often located within regions containing multiple overlapping promoter-like sequences, as well as additional promoter sequences [12,14,15]. These putative or functional promoter signals might also play a regulatory role which can be negative if those sites compete with each other, or positive if they help in attracting the RNAP to the functional promoter [16]. However, initiation of the transcription process is influenced not only by the promoter DNA sequence or presence of promoter-like sequences, but also by a variety of different factors, including proteins serving as repressors or activators, small ligands, temperature, solution composition and concentration, and other parameters [17].

Inhibition of transcription initiation is an important and common mechanism of gene expression regulation in bacteria. Proteins acting as repressors bind to specific sites, called operators, often positioned within or in close proximity of the promoter region. The level of gene expression is modulated as a result of steric hindrance or interactions between the transcription factor bound to these sites and RNA polymerase [18,19,20]. Promoter architecture, as well as relative positioning of the operator and the promoter, dictates the nature of these interactions and defines a precise mechanism of repression [21,22,23]. Transcriptional repressors can act on any of the initiation steps, beginning with acting as a steric hindrance to RNAP binding, through inhibition of open complex formation and stability, and ending on prevention of promoter clearance.

One of examples where negative transcription regulation is a crucial strategy for precise gene regulation is illustrated by the toxin-antitoxin (TA) systems [24,25,26]. These systems are formed by small modules, usually composed of a pair of genes coding for a toxin which affects one of the vital cellular processes, and a cognate antitoxin. TA cassettes are widely distributed on plasmids and chromosomes of many bacterial and archaeal species [27,28,29]. The toxic components of TA systems can be regarded as intracellular molecular bombs whose liberation induces bacterial cell death or growth arrest [25,30]. Currently, the TA systems are divided into six types based on the nature and mechanism of action of the specific antitoxin [27,31]. The study presented here focuses on class II TA, which is the most numerous and best studied type to date. In this type, a long-lasting toxin and a short-lived antitoxin are proteins that interact with each other producing a non-toxic complex. To ensure a secure balance between them, both proteins are typically co-expressed from a common promoter that is negatively autoregulated by the TA complex [24,25,26]. The antitoxin, which is encoded as the first gene in the operon, is a sequence-specific DNA binding protein, recognizes an operator site that overlaps with the TA operon promoter and functions as a weak repressor. The toxic protein typically is encoded downstream of the antitoxin gene, and usually acts as a transcriptional corepressor that remodels and stabilizes the antitoxin structure and its binding to DNA or in some cases, also as a promoter de-repressor, depending on the toxin-antitoxin ratio. Such a phenomenon where toxin-antitoxin complexes with different stoichiometry of proteins have different affinity to the operator site is called “conditional cooperativity” [32,33,34]. When the ratio of toxin to antitoxin is in favour of the latter, then a weak repression of the operon is observed. Under conditions of balanced expression of both proteins, full operon repression is obtained. However, in a situation when a toxin is produced in excess over the antitoxin, the TA complexes of different stoichiometry are formed and de-repression of the operon occurs [32,35]. This mechanism is supposed to prevent accidental toxin activation and enables quicker recovery of translation after toxin’s overabundance [36]. The majority of type II TA modules follows the general pattern of transcription autoregulation described above, however, some exceptions to this rule have been also described. There are tripartite TA cassettes where an additional gene encodes a protein serving as a transcriptional regulator, systems with the reverse order of toxin and antitoxin genes in the TA operon (both having their own promoters), examples where additional regulatory elements like promoters were found within the TA unit, modules where antitoxin or the toxin serve as the only repressors of the system or finally TA loci which do not undergo autorepression but instead are controlled by the global regulator [25,26].

The *yefM-yoeB* cassette has been identified on *E. coli* chromosome and also in the genomes of many diverse bacterial species. Moreover, its homolog named *axe-txe* has been discovered on different *Enterococcus faecium* plasmids. Similarly to a typical type II TA system, the *E. coli yefM-yoeB* module undergoes transcriptional autoregulation where the YefM antitoxin serves as a weak repressor while YoeB acts as a co-repressor enhancing efficiency of promoter inhibition [37,38]. To date, only three other *yefM-yoeB* systems were assessed in terms of their gene regulation and these investigations showed that in other organisms this process can be more complex than in the *E. coli* example. Expression of this locus in *Streptococcus pneumoniae* was shown to be driven from two different promoters located upstream of the antitoxin gene, one of them being regulated by the YefM-YoeB proteins while the other, weaker promoter is constitutive [39]. Even more complicated regulation pattern was described for the *yefM-yoeB* system from *Lactobacillus rhamnosus* and for *axe-txe* from pRUM plasmid of *E. faecium,* where additional promoters were identified within the antitoxin gene and an antisense transcript was found to overlap with the toxin gene [40,41].

**Figure 1:**
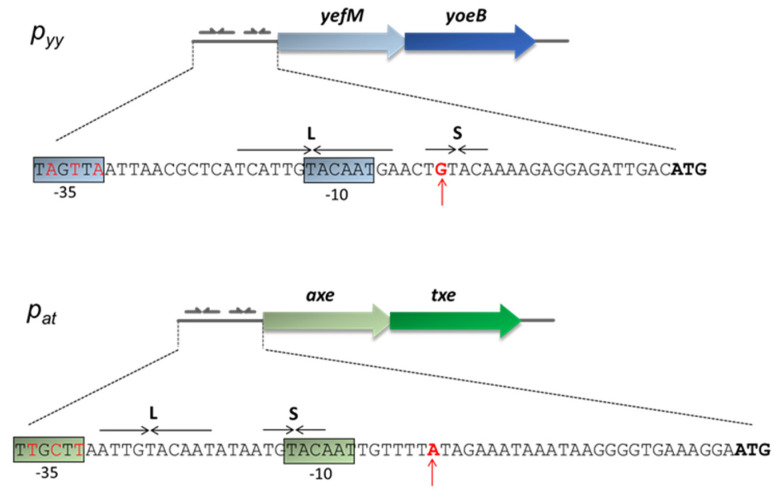
Nucleotide sequence of the *p_yy_* and *p_at_* promoter regions. Transcription start-site is marked by a red vertical arrow. Hexameric −10 and −35 promoter motifs are color boxed. YefM and Axe ATG start codons are in bold. Long (L) and short (S) palindromic repeats recognized by the antitoxins and serving as repressor binding sites are denoted by horizontal arrows.

This paper focuses on the *Escherichia coli* K-12 chromosomal *yefM-yoeB* locus and the *axe-txe* cassette encoded by the pRUM plasmid of *Enterococcus faecium* [42]. The corresponding antitoxins (Axe and YefM) and toxins (Txe and YoeB) share ~25% and ~50% of protein sequence identity, respectively. Crystal structure of the YefM-YoeB complex shows that it is a heterotrimer composed of one toxin and two antitoxins monomers [43]. For Axe-Txe, only molecular modelling and protein-protein docking were performed to date, however, a very high structure similarity to the YefM-YoeB complex was found [44,45]. Nevertheless, in terms of their structure, the proteins of both modules had diverged sufficiently enough to interact productively only with their cognate partners [44]. Transcriptional autoregulation of the *yefM-yoeB* and *axe-txe* operons is mediated by binding of the N-terminal domains of the antitoxin molecules to pairs of palindromes that overlap their cognate promoter (Figure 1). The sequences and spacing of these palindromes are maintained in many *yefM-yoeB* cassettes in diverse bacteria, including the enterococcal *axe-txe* module [37].

The aim of this study was based on an observation that transcriptional activity and repression level of *p_yy_* and *p_at_* promoters are substantially different when assessed in vivo in *E. coli* cells. Thus, unlike the *p_yy_* promoter which is weaker and fully repressed by YefM-YoeB, the *p_at_* promoter is extremely strong and is not efficiently inhibited by Axe-Txe [37,41]. Thus, the goal of this report was to establish molecular bases of these discrepancies. With a series of in vivo, as well as in vitro experiments, we show that the differences in the activity of these two investigated promoters result from a disparate −35 promoter element, while variations in their repression level arise from distinct locations of the repressor binding sites within the corresponding promoter regions. This determines that in each case a different step in transcription initiation is affected by the TA complexes. Moreover, we identified an additional promoter upstream of *p_at_* which contributes to the overall transcriptional activity of the *axe-txe* unit. We have decided to perform majority of our experiments with the well-defined model organism—*E. coli* firstly because it is a natural host of the YefM-YoeB system and secondly, the activity of in vivo transcriptional fusions used for enterococcal cells are very low which makes it difficult to discern subtle differences. Nevertheless, the activity of *p_at_ and p_at2_* promoters was also confirmed in the enterococcal cells. This study provides further evidence for the existence of an additional layer of complexity in transcriptional regulation of the TA systems which is often overlooked.

## 2. Results

### 2.1. Differences in the −35 Box Sequence are Responsible for Different Strengths of the p_at_ and p_yy_ Promoters

Analysis of the *p_at_* and *p_yy_* promoter sequences shows a significant degree of homology within the main promoter and operator elements (Figure 1). Both promoters have an identical −10 box (5′-**TA**cAA**T**-3′) which differs from the consensus sequence (5′-**TA**tAA**T**-3′) at position −10 where there is a C instead of T, however, that position is the least conserved site in this region [5]. In the consensus sequence for the −35 motif, the first three bases are the most conserved among promoters (5′-**TTG**ACA-3′) [5]. In case of *p_yy,_* the second T is replaced by A (5′-**TaG**ttA-3′), while for *p_at_* all three nucleotides are the same as in the consensus sequence (5′-**TTG**ctt-3′). In both promoters, the linker between −10 and −35 boxes has a length of 17 bp which is typical for most promoters. Regarding the transcription start site, there is a purine base in both promoters, A in *p_at_* and G in *p_yy_*.

The operator region located within both promoters consists of long (L) and short (S) repeats which provide a binding site for the antitoxins, YefM or Axe. Both palindromes have a common 5′-TGTACA-3′ core motif separated by the same distance of 12 bp from center to center (Figure 1). This distance is precisely maintained in the regulatory regions of *yefM-yoeB* in genomes of many diverse bacterial species, suggesting that it is a key feature of *yefM-yoeB* transcriptional control [37].

**Figure 2:**
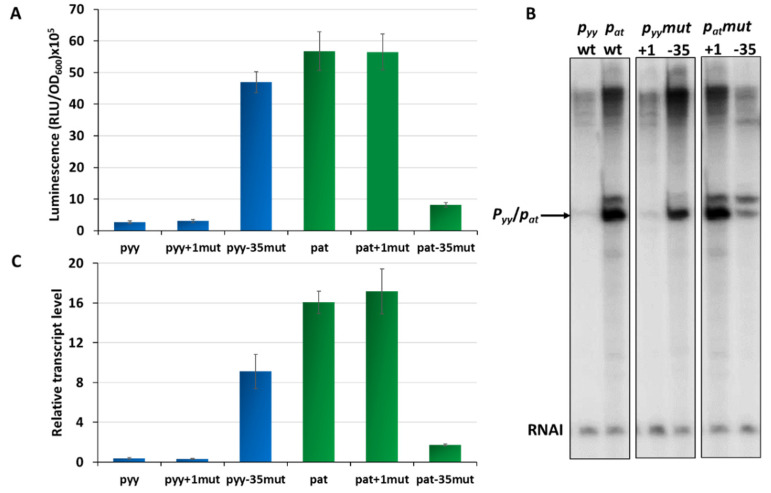
Transcriptional activity of *p_yy_* (blue bars) and *p_at_* (green bars) promoters and their derivatives. (**A**) Transcriptional fusions of wild-type *p_yy_* and *p_at_* promoters and their +1 position and −35 box swapped mutants. Appropriate pBBRlux vector fusions were introduced into the SC301467 strain. Luminescence was measured in RLU (relative luminescence units) normalized to OD, when cell cultures reached OD_600_~0.5. The basal activity of pBBRlux-amp vector control was ~100 units. These results are average of at least three independent experiments, error bars represent standard deviation (S.D.). (**B**) Representative image of in vitro transcription activity of *p_yy_* and *p_at_* promoters and their +1 position and −35 box swapped mutants. Multiround in vitro transcription experiments were performed using *E. coli* σ^70^ RNA polymerase holoenzyme (25 nM) and pTE103 DNA templates containing appropriate promoter fragments (5 nM). Reactions were performed and analyzed as outlined in Materials and Methods. All lanes that are shown originated from the same gel. (**C**) Relative transcript level obtained in in vitro transcription experiments were normalized to the RNAI level. Experiments were done at least in triplicate. Error bars represent S.D.

Our previous studies on the *p_at_* and *p_yy_* promoters demonstrated that they may have quite different transcriptional activities [37,41]. However, for *p_yy_* experiments a *lacZ* reporter fusion was used, while for assessing *p_at_* promoter activity a *lux* operon fusion was applied. This makes a direct comparison of the activity of both promoters impossible. To unify experimental conditions, here we employed *p_yy_* and *p_at_* transcriptional fusions with a promoterless *lux* operon as a reporter, using the pBBRlux-amp vector. pBBRlux-pyy and pBBRlux-pat constructs were introduced into *E. coli* SC301467 strain which is a MG1655 derivative where 5 out of 13 known type II chromosomal toxin-antitoxin cassettes have been deleted, including *yefM-yoeB*, to avoid any possible cross-interactions with homologous systems [46]. Luciferase activity was measured in RLU (relative luminescence units) when the cell culture reached OD_600_~0.5 and the results were divided by optical density of the cultures. The *p_at_-lux* fusion produced over 5 × 10^6^ RLU, while *p_yy_*-*lux* fusion gave an activity below 3 × 10^5^, which is over 20 times less than the other fusion (Figure 2A). To verify these results, we performed multiround in vitro transcription reactions. For this purpose, pTE103 plasmid derivatives (pTEpat and pTEpyy) were used as templates. These constructs contain a strong T7 transcription terminator region downstream of the cloned promoter fragment and produce the RNAI transcript of 108 nt which serves as an internal control of the transcription process. In these experiments, *p_yy_* turned out to be about 40 times transcriptionally weaker than *p_at_* (Figure 2B,C).

Overall, both the in vivo and in vitro results clearly showed that transcription initiating from *p_yy_* is significantly less productive than from the *p_at_* promoter.

Next, we asked which elements in the promoter sequences may be responsible for such a significant difference in their activity. Based on our *in silico* analysis of *p_yy_* and *p_at_* promoter sequences we decided to evaluate the effects of the transcription start site nucleotide and the −35 box which are different in both promoters. To achieve that we designed constructs with pBBRlux and pTE103 vectors in which the +1 and −35 region of both promoters have been swapped. By using site directed mutagenesis, we inserted G instead of A at the transcription start site of the *p_at_* promoter, while in the same position of *p_yy_* G was replaced by A. In this way we obtained constructs pBBRlux_pat+1mut and pBBRlux_pyy+1mut. Similarly, we also changed the *p_at_* −35 box from TTGCTT to TAGTTA, and *vice versa* for the *p_yy_* promoter, thus obtaining the pBBRluxpat-35mut and pBBRluxpyy−35mut plasmids. All of these pBBRlux derivatives were introduced into *E. coli* SC301467 strain and luciferase activity was measured at mid-exponential phase and calculated as described above. Swapping of +1 positions did not cause any substantial difference in the overall transcriptional activity of both investigated promoters, while swapping of the −35 element boxes resulted in a drastic change (Figure 2A). The same modifications were made in the pTEpat and pTEpyy plasmids which subsequently were used as templates in in vitro transcription experiments (Figure 2B,C). The −35 mutated *p_yy_* promoter had an 18- and 23-times increased activity when compared to wild type *p_yy_*, in vivo and in vitro respectively, while the −35 swapped *p_at_* promoter was severely weakened when compared to wild type *p_at_* and exhibited a 7- and 9-times decreased activity, in vivo and in vitro respectively.

These in vivo, as well as in vitro results undoubtedly indicate that the −35 promoter box sequence is a major element responsible for differences in the activity of both promoters.

### 2.2. Axe-Txe and YefM-YoeB Repressor Complexes Exhibit Similar Specificity to Cognate and Non-Cognate Operator Sequences

Phylogenetic analyses had revealed that distantly related TA systems generally do not display cross-talk between their protein counterparts, whereas close homologs are often able to cross-interact [47]. In our previous publication we established that despite the fact that the *E. coli* chromosomal *yefM-yoeB* system and enterococcal plasmid *axe-txe* module are highly homologous, their proteins do not cross-interact. We have also demonstrated that a single amino acid substitution, Asp83Tyr, in Txe allows this toxin to efficiently interact with the YefM antitoxin [44]. However, an issue regarding specificity of the repressor complex to the homologous operator regions has not been investigated so far.

**Figure 3:**
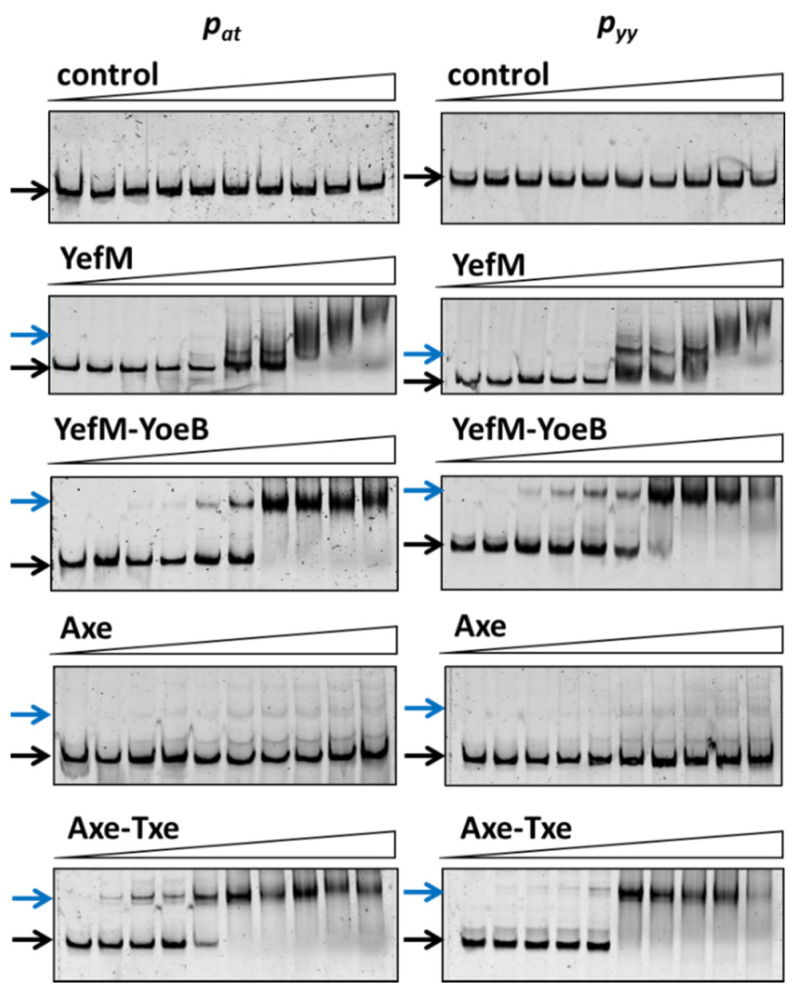
Antitoxin, YefM and Axe, as well as toxin-antitoxin complexes’, YefM-YoeB and Axe-Txe, binding to the *p_yy_* and *p_at_* promoter-operator regions. 25 ng of cy5-labeled double stranded DNA fragments encompassing *p_yy_* and *p_at_* promoter-operator regions were subjected to EMSA with increasing amounts of bacterial cell lysates (0; 0.05; 0.1; 0.2; 0.4; 0.9; 1.8; 3.7; 7.4; 14.8 µg). Reactions were incubated for 20 min at RT, separated by native 6% PAGE and processed further as described in Materials and Methods. Black and blue arrows denote positions of unbound DNA and protein-DNA complexes, respectively.

Here, EMSA experiments were performed to test the affinity of antitoxins and the toxin-antitoxin complexes of both investigated cassettes to their own and to the cognate operator sequences. For this purpose, BL21(DE3) crude extracts were prepared where YefM, YefM-YoeB, Axe and Axe-Txe were overproduced from appropriate pET22(b) derivatives (Figure S1). BL21 cells, like other *E. coli* B strains, do not possess the chromosomal *yefM-yoeB* module, and thus any potential cross-interactions between homologous systems can be excluded [43]. However, in this experimental set-up we can only compare the relative binding of each protein or protein complex to both investigated promoter fragments, since the level of protein overproduction in each of the used cell lysates was different (Figure 3).

**Figure 4:**
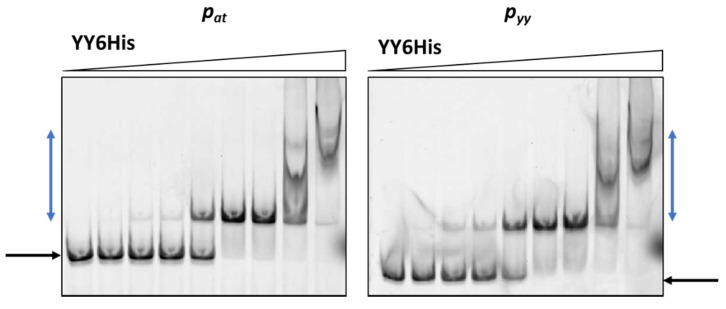
Purified YefM-YoeB complex binding to *p_yy_* and *p_at_* promoter fragments assessed by EMSA. 10 nM of cy5-labeled ds DNA fragments were subjected to EMSA with increasing amounts of purified YefM-YoeB6His protein complex (0, 10, 25, 50, 100, 200, 400, 800, 1600 nM). A representative gel is shown. Black and blue arrows denote positions of unbound DNA and protein-DNA complexes, respectively.

Cy5-labeled DNA fragments containing promoter/operator region of *p_yy_* and *p_at_* were incubated with increasing concentrations of cell lysates. As a control, crude extract of cells with pET22(b) vector plasmid was used and in this case no binding was observed (Figure 3). The YefM antitoxin bound to the operator fragment of both promoters at higher amounts of the lysate (beginning at 0.9 µg), giving smeary bands that indicate unstable DNA binding (Figure 3). The Axe-DNA complexes at both promoters were visible only as very faint bands. This could be due to lower Axe concentration in the crude lysate (Figure S1). On the other hand, both toxin-antitoxin complexes showed efficient and stable DNA binding to both DNA fragments. First YefM-YoeB-DNA complexes were formed at 0.1 µg of lysate, while the whole DNA was shifted at 1.8 µg of the lysate for both promoters. For Axe-Txe-DNA complexes, first of them could be detected even 0.05 µg of the protein lysate while the whole DNA was shifted at 0.9 µg of the used lysate, similarly for both promoters (Figure 3). This is in agreement with our previous observation that YefM-YeoB and Axe-Txe complexes bind to their promoter fragments more efficiently than the antitoxins alone [37,41]. However, what is important to mention, each of the investigated proteins or protein complexes bound more or less equally well to its own as to the homologous promoter fragment, although the protein complexes seem to be slightly more potent towards their native promoters. To verify this observation, we repeated the EMSA experiment with a purified YefM-YeoB6His complex (Figure 4). In these experiments first protein-DNA complexes appeared at 25 nM of YefM-YoeB, full DNA shift could be detected at 200 nM, while additional higher order complexes started to form at 800 nM of YefM-YoeB concentration for both cognate promoters. Similarly, to experiments with crude extracts, these EMSA results demonstrated that the YefM-YoeB complex has approximate affinity to both tested templates (Figure 4).

These data clearly indicate that proteins of the *axe-txe* and *yefM-yoeB* systems are able to bind to their cognate and non-cognate operator regions with similar efficiency.

### 2.3. YefM-YoeB Complex Represses Transcription at the p_at_ and p_yy_ Promoters with Different Efficiency

**Figure 5:**
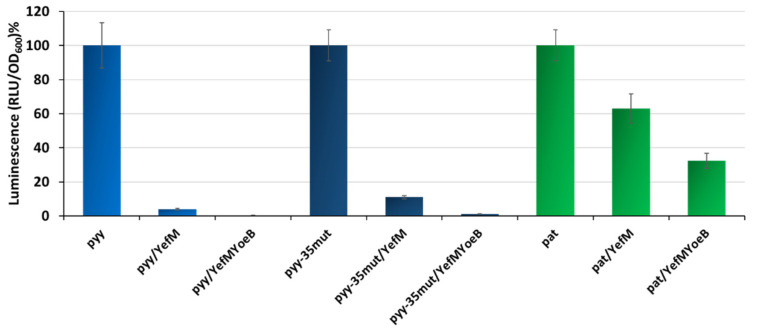
Transcription repression of *p_yy_*, *p_yy_*−35mut and *p_at_* mediated by YefM and YefM-YoeB in vivo. Transcriptional *lux* gene fusions of wild-type *p_yy_, p_yy_*−35mut, and *p_at_* promoters were made in pBBRlux-amp vectors and were introduced into the SC301467 strain together with pBAD33 vector control (for promoter unrepressed conditions—first bars) and pBAD33 derivatives producing YefM or YefM-YoeB complex. Assays were conducted after induction with 0.2% L-arabinose. Luminescence was measured in RLU (relative luminescence units) when cell cultures reached OD_600_~0.5. The basal activity of pBBRlux-amp vector control was ~100 units. These results are average of at least three independent experiments. Error bars represent standard deviation (S.D.) Results are normalised separately for each promoter.

Our previous studies showed that *p_yy_*-mediated transcription is efficiently inhibited by the YefM-YoeB complex, almost reaching the background level, while *p_at_* repression by Axe-Txe is not efficient and a substantial activity can be still detected in vivo [37,41]. However, as mentioned before, the *p_yy_* experiments were done with the use of a *lacZ* reporter gene fusions, with YefM or YefM-YoeB complex supplied *in trans* from a pBAD33 plasmid, whereas for *p_at_* studies *in cis* transcriptional fusions with the use of pBBRlux vector were employed. This again makes a direct comparison of the effectiveness of both systems impossible. To overcome this problem, here we introduced *p_at_* and *p_yy_* promoter fusions with the *lux* gene (employing a pBBRlux plasmid) into the SC301467 strain, and co-transformed them with pBAD33 derivatives overexpressing YefM and YefM-YoeB under L-arabinose inducible promoters. The activity of *lux* fusions was measured when bacterial cell cultures reached OD_600_~0.5. In this set of experiments, we also included the *p_yy_* promoter variant with −35 box mutated to that of *p_at_*. The reason for this was to have a stronger version of the *p_yy_* promoter as a control, i.e., to assess if the potential diversity between *p_yy_* and *p_at_* is not solely due to the difference in the RNA polymerase affinity to a weaker or stronger promoter sequence. The results obtained are presented in Figure 5; data was normalized to relative percentage units to enable comparisons between promoters that demonstrate substantial diversity in basal activity. It is evident that the YefM overexpression alone had a much stronger effect on *p_yy_* and *p_yy_*−35mut, than on *p_at_*: YefM lowered the *p_yy_* promoter activity to 4% and that of *p_yy_*−35mut to 11%, while *p_at_* transcriptional activity was repressed to only 63% relative to control conditions. Moreover, the YefM-YoeB complex abolished activity of both *p_yy_* and *p_yy_*−35mut to almost background levels, in both cases to below 1%, while the activity of *p_at_* was still detected at a relatively high level of 32% (Figure 5).

**Figure 6:**
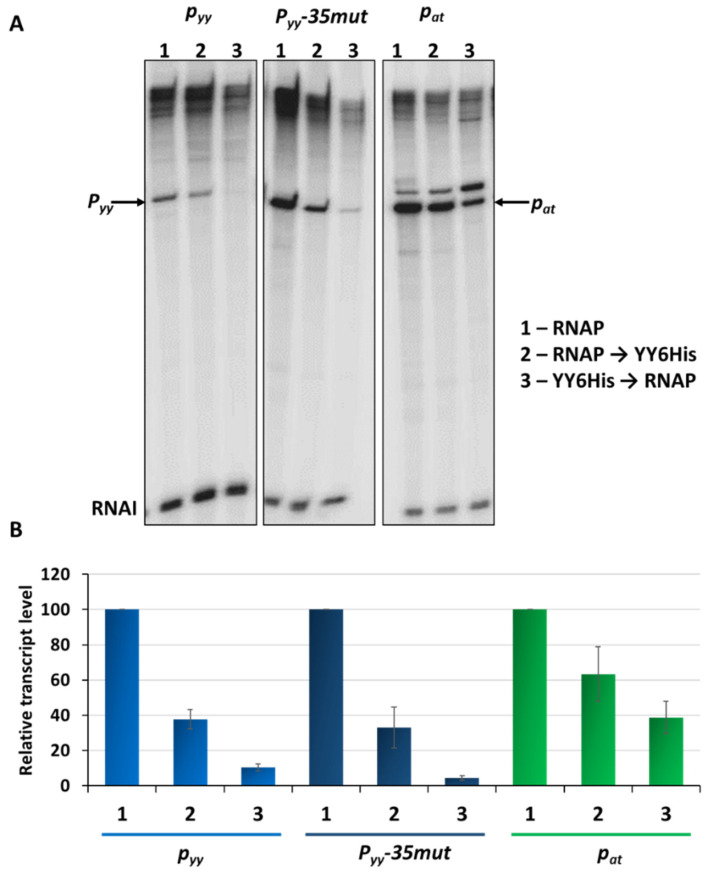
Transcription repression mediated by YefM-YoeB in vitro. (**A**) Multiround in vitro transcription experiments were performed using 25 nM *E. coli* σ^70^ RNA polymerase holoenzyme and 5 nM pTE103 DNA templates containing indicated promoter fragments. Reactions were performed with RNAP alone (lanes marked as 1), or with 200 nM YefM-YoeB6His complex that was added before (lanes marked as 2) or after RNAP binding (lanes marked as 3), as indicated. Reactions were started by the addition of nucleotides, and then processed and analyzed as outlined in Materials and Methods. All lanes that are shown came from the same gel. (**B**) Relative transcript level obtained in in vitro transcription experiments was normalized to the RNAI level. Experiments were done in triplicate. Error bars represent S.D. Results are normalised separately for each promoter.

In parallel, in vitro transcription experiments were performed. First, a control experiment was carried out where the assay conditions were adjusted to the weakest promoter to be tested. For this purpose, 5 nM pTEpyy DNA template was used in multiround in vitro transcription in which 25 nM *E. coli* RNAP holoenzyme and different concentrations of YefM-YoeB6His complex were added to assess a proper repressor concentration to get the best repression conditions (Figure S2). Total inhibition of *p_yy_* activity was observed within the range of 100 to 200 nM of the YefM-YoeB6His complex. Then, in vitro transcription assays were performed using pTE103 derivatives with *p_yy_*, *p_yy_*−35mut and *p_at_* inserts, as templates. RNA polymerase holoenzyme, as well as the YefM-YoeB6His complex, were added in variable order to monitor the effectiveness of transcription repression. As can be seen in Figure 6, the most efficient transcription inhibition for all three promoters is observed when the TA complex was allowed to bind DNA before RNAP was added. Under this set-up, transcription from *p_yy_* was abolished to the level below 10%, regardless whether the weaker wild type or stronger *p_yy_*−35mut promoters were used as templates. However, *p_at_* was not as efficiently inhibited, with transcript level reaching 38% of that detected in the absence of YefM-YoeB6His (Figure 6).

Both, the in vivo and in vitro experiments indicate that transcriptional activity of the *p_yy_* promoter is much more efficiently repressed when compared to the *p_at_* promoter, regardless of the basal promoter strength.

### 2.4. Different Repression Level of p_yy_ and p_at_ May Result from Repressor Acting on Different Steps in the Transcription Initiation Process

Next, we raised a question why the efficiency of transcription repression in the case of *axe-txe* system is not as effective as that mediated by the *yefM-yoeB* cassette, in a situation when both antitoxins and TA complexes demonstrate similar affinity to their own and cognate operator DNA. We looked closer at the sequences of both promoters and noticed that although the sequences of the operator sites are identical in their core, their relative location within the promoter region differs (see Figure 1). In case of *p_yy_* the primary repressor binding site—the L palindrome, covers the whole −10 box and the second repeat, the S palindrome, surrounds the transcription start site. In contrast, in case of *p_at_* the L repeat is located within the linker between the −10 and −35 boxes, whereas the S repeat only partially overlaps the −10 hexamer. Such different placement of repressor binding sites within the promoter may potentially cause a different response to the repressor protein. The first situation seems to be the case of a classic steric hindrance when repressor completely blocks binding of the RNA polymerase to the promoter region. In the second case, there is a possibility that both proteins can bind to the promoter simultaneously and then RNAP can either remove the bound repressor or one of the subsequent transcription initiation steps is hindered, such as the open complex formation or promoter escape.

**Figure 7:**
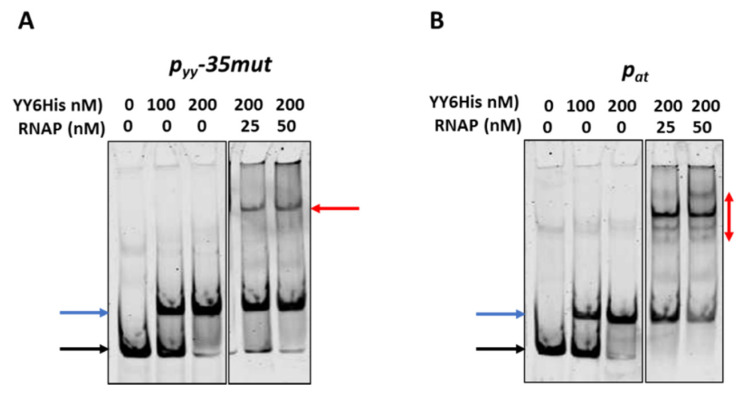
Competitive EMSA on *p_yy_−35mut* (**A**) and *p_at_* (**B**) promoter fragments. 10 nM cy5-labeled ds DNA fragments (225 bp) were subjected to EMSA with indicated amounts of purified YefM-YoeB protein complex and *E. coli* RNAP holoenzyme. First YefM-YoeB complex was bound to DNA for 15 min, next RNAP was added and binding was extended to another 15 min. After incubation, samples were run on a 4% native PAGE and analyzed as outlined in Materials and Methods. Black and blue arrows denote positions of unbound DNA and the YY6His-DNA complexes, respectively. Red arrows indicate RNAP-DNA or possible RNAP-YY6His-DNA complexes. Representative gels are shown. All lanes that are displayed side by side came from the same gel.

To investigate this issue further we first performed competitive EMSA experiments. In these assays 10 nM of ~225 bp cy5-labelled DNA fragments encompassing promoter/operator region of *p_yy_−35mut* and *p_at_* were subjected to interactions with the YefM-YoeB6His complex; where indicated, RNAP was also added. Representative EMSA gels are depicted in Figure 7. As can be noticed, approximately all promoter DNA is bound by the TA complex at 200 nM YefM-YoeB. As the concentration of RNAP increases, the band corresponding to the DNA-YefM-YoeB complex decreases in intensity in comparison to the lane with only the repressor being bound, and this effect is much more pronounced in the case of the *p_at_* promoter than the *p_yy_−35mut* promoter (Figure 7, blue arrows). Moreover, in the lanes with RNAP and repressor present, multiple DNA-protein complexes are formed in the case of *p_at_* promoter, while only a single band is observed in lanes with *p_yy_* samples (Figure 7, red arrows). It suggests that YefM-YoeB binds to the *p_yy_* promoter variant in a way that RNAP cannot displace it very easily, while at *p_at_* its binding position enables the RNA polymerase to also bind simultaneously or to clear it away more efficiently.

To test our hypothesis further, DNaseI footprinting experiments were performed where we aimed to evaluate differences in the region covered upon RNAP and YefM-YoeB binding when added separately or in competition. First, YefM-YoeB6His titration was done to assess optimal concentration of the complex to be used and to confirm its proper binding. For this purpose, 20 nM cy5-labeled promoter/operator DNA fragments of *p_yy_*, *p_yy_−35mut* or *p_at_* and increasing concentrations of YefM-YoeB complex (ranging from 0.25 to 2 µM) were incubated together for 10 min, and then reactions were treated with the DNaseI endonuclease. Fully developed footprints overlapping appropriate L and S palindromic repeats could be observed for all three tested templates at 0.5 µM TA complex concentration (Figure S3).

**Figure 8:**
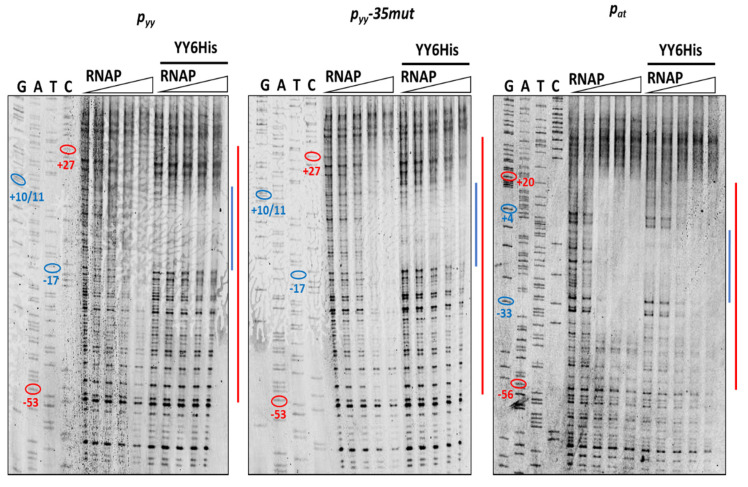
DNaseI footprinting on *p_yy_* wt, *p_yy_−35mut* and *p_at_* promoter-operator regions. Footprinting reactions were performed as outlined in Materials and Methods using increasing concentrations of *E. coli* RNA polymerase holoenzyme (0, 50, 100, 150, 200 nM) bound to 20 nM cy5-labeled appropriate DNA fragment for 10 min at 37 °C (left panels of the gels) or promoter fragments were first incubated with 1 µM YefM-YoeB6His complex for 10 min at 37 °C and then with increasing concentration of RNAP (right panels)—as indicated above the gels. Next, the samples were treated with DNaseI and reactions were run on 8% denaturing acrylamide gel along with sequencing reactions (GATC). The relative positions of regions on the upper strand that are protected from DNaseI digestion by the YefM-YoeB complex or by RNAP are illustrated at the right side of the gels by the blue and red lines, respectively. On sequencing reactions lines ovals and numbers denote positions relative to the transcription start site.

Next, we performed DNaseI footprinting experiments with RNAP alone and with both, YefM-YoeB and RNA polymerase holoenzyme, again using all three promoter DNA templates. In the reactions with YefM-YoeB, first the TA complex (at 1 µM concentration) was allowed to bind to the DNA and then different concentrations of RNAP were added. To stabilize the RNA polymerase interactions with promoter fragments, the first two ribonucleotides were included in the reaction mixtures. It has been previously shown that binding of the initiating NTPs helps to shift the closed RNAP-DNA complex to the more stable open complex. RNAP alone covered the promoter region extending from around −55 do +25 base pairs (Figure 8—red lines). When YefM-YoeB complex was bound first to both versions of the *p_yy_* promoter/operator sequence, subsequent addition of RNAP did not produce any further footprint, and only footprint corresponding to the repressor was visible (Figure 8—blue lines). On the contrary, when YefM-YoeB was bound to the *p_at_* promoter and then increasing concentrations of RNAP were added, the footprint corresponding to the TA repressor has successively disappeared and extended to the one produced by the RNAP holoenzyme (Figure 8). This means that in the case of the *p_yy_* promoter, the TA repressor complex strongly binds to the operator and RNAP cannot clear it away, whereas at the *p_at_* promoter RNAP can bind the DNA at the same time as repressor or is able to remove the bound repressor complexes from their sites.

### 2.5. Additional Active Promoter that Contributes to the Overall Transcriptional Activity of the Axe-Txe Cassette Is Identified Just Upstream of p_at_

**Figure 9:**
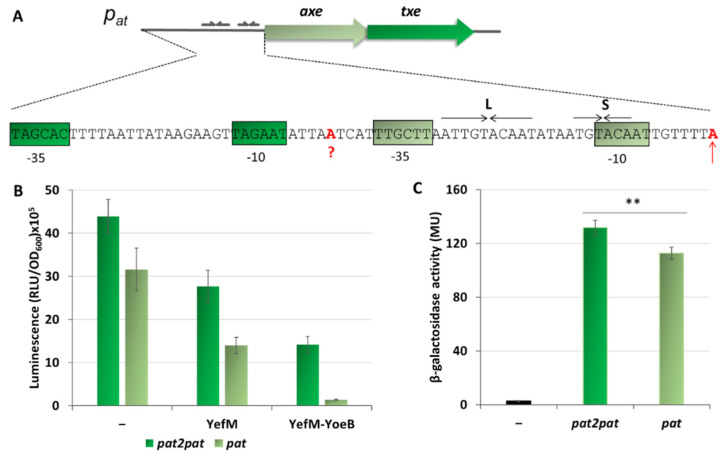
Contribution of an additional promoter located upstream of *p_at_* to the overall *axe-txe* transcription. (**A**) Nucleotide sequence of the region upstream of *p_at_* transcriptional start site (marked in red and indicated by vertical arrow). Putative *p_at2_* start site is marked in red and question mark. The *p_at_* and *p_at2_* −10 and −35 hexamers are shown in gray-green and green boxes, respectively. (**B**) pBBRlux vector fusions with promoter fragments containing both promoter sequences (*pat2pat*) or only the major *p_at_* promoter (*pat*) were introduced into *E. coli* SC301467 strain together with pBAD33 derivatives bearing the *yefM* or *yefM-yoeB* genes under arabinose inducible promoter. (–) indicates vector control. Expression of *yefM* or *yefM-yoeB* was induced by addition of 0.2% L-arabinose at a time of inoculation. Luminescence was measured in RLU (relative luminescence units) when cells reached OD_600_~0.5. (**C**) pTCVlac vector fusions with promoter fragments containing both promoter sequences (*pat2pat*) or only the major *p_at_* promoter (*pat*) were introduced into OG1RF *Enterococcus faecalis* strain. (−) indicates strain with pTCVlac plasmid without any fusion. β-galactosidase activity was measured in MU (Miller units) when cells reached OD_600_~0.5. Experiments were done in triplicate. Error bars represent S.D. *p*-value calculated for panel C is indicated (** *p* < 0.01).

**Figure 10:**
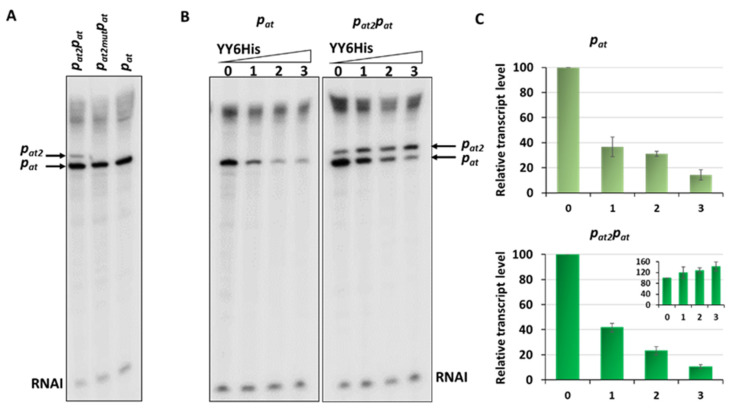
In vitro transcription with DNA containing different fragments of the *p_at_* promoter. Multiround in vitro transcription experiments were performed using 25 nM *E. coli* σ^70^ RNA polymerase holoenzyme and 5 nM pTE103 DNA templates containing indicated promoter fragments. Reactions were performed with RNAP alone or with addition of increasing concentrations of the YefMYoeB6His complex, as indicated. Reactions were started by the addition of nucleotides, and then processed and analyzed as outlined in Materials and Methods. (**A**) In vitro transcription with DNA fragment where both *p_at_* promoters are present—*p_at2_p_at_*, with *p_at2_* promoter mutated—*p_at2mut_p_at_* and with only *p_at_* promoter. (**B**) Transcription repression mediated by YefM-YoeB in vitro. 0—without YY6His, 1—200 nM YY6His, 2—400 nM YY6His and 3—800 nM YY6His. All lanes that are shown came from the same gel. (**C**) Relative transcript level obtained in in vitro transcription experiments, normalized to the RNAI level. The *p_at_* transcript level is presented on the big graphs, while the calculated level of *p_at2_* transcript is shown on the inset graph. Experiments were done at least in triplicate. Error bars represent S.D. Results are normalised separately for each promoter.

On the in vitro transcription gels, we have noticed the appearance of an additional band just above the transcript derived from the *p_at_* promoter (Figure 2B and Figure 6A). After careful analysis of the nucleotide sequence upstream of *p_at_* we found a potential promoter sequence (Figure 9A). This finding was also confirmed by the PromScan *in silico* analysis. To verify if this DNA sequence somehow influences transcriptional activity of the main *p_at_* promoter in vivo, we made pBBRlux constructs where we cloned the *p_at_* promoter without this additional upstream fragment. The results are depicted in Figure 9B. Activity of the *p_at_* in construct devoid of the upstream promoter, *p_at2_* as we called it, was 28% lower in comparison with the sample where both promoters were present. To confirm that we have identified this promoter correctly, in vitro transcription assays have been also performed. Apart from previously used pTEpat possessing *p_at2_p_at_* fragment, we have cloned DNA fragments containing the main *p_at_* promoter only (pTEpat1) and *p_at2_p_at_* in which −10 box of the *p_at2_* promoter was disrupted by changes **TA**GAAT to **CG**GAAT (pTEpat2mut−10pat) into pTE103 vector. Our results undoubtedly show that the upper band is not present when the DNA template contains mutated or no *p_at2_* sequence (Figure 10A). Our calculations revealed that the band corresponding to *p_at2_* transcript represents 16% ± 3 of the main *p_at_* transcript level.

In addition, since the Gram+ RNA polymerases in comparison to Gram− RNAP may exhibit a decreased stability at weak promoters, and that some promoter signals recognised by *E. coli* RNAP may not be transcribed by enterococcal RNAP [48], we decided to check the activity of both promoter regions in *Enterococcus faecalis* cells. We have cloned *p_at_* and *p_at2_p_at_* promoter fragments into pTCVlac shuttle vector as a transcriptional fusions, with the *lacZ* gene possessing Gram+ ribosome binding site. These experiments clearly indicate that the *p_at2_* promoter contributes to the transcriptional activity of this region, as the fragment with both promoters present has an activity that is 15% higher than the *p_at_* promoter alone (Figure 9C).

Next, to assess the repression efficiency of both promoter fragments in vivo, similar experiments were performed as described above (Figure 5), where expression of the antitoxin and the YefM-YoeB complex were induced from an L-arabinose dependent promoter in pBAD33 derivatives. The results presented in Figure 9B demonstrate that after removal of the *p_at2_* promoter sequence, the observed repression efficiency of the major *p_at_* promoter was greatly improved and reached ~95%, however, it still remained at a relatively high level of around 140 thousand RLU units. As a control, we have also done the same kind of experiment with *p_at_−35mut* promoter fusion, a weaker version of *p_at_* promoter (Figure S4). From these results it is clear that irrespective to the promoter strength, *p_at_* is less efficiently repressed, both by the antitoxin alone and by the TA complex, in comparison to the *p_yy_* promoter. Moreover, the difference in the unit number between constructs with *p_at_* and *p_at_p_at2_* was around 1,300,000 RLU units and persisted upon YefM or YefM-YoeB overproduction. This could suggest that the *p_at2_* activity is not influenced by the repressor or repressor complex in vivo.

To further our investigations of the contribution of the newly identified *p_at2_* promoter to the transcription activity and repression efficiency of the main *p_at_* promoter additional in vitro transcription assays were performed. For these experiments, DNA templates in pTE103 with both promoters or with only the *p_at_* promoter were used. The results depicted in Figure 10 first of all show that the inhibition of the main promoter by different concentrations of YefM-YoeB6His complex is approximately similar on both tested templates. Moreover, we have observed that for the construct with both promoters present, the more efficient repression of *p_at_*, the higher *p_at2_* transcript level (Figure 10). These experiments again confirmed that the repression of the *p_at_* promoter is not as efficient as that of *p_yy_*, regardless of its strength (compare Figure 10B and Figure S2).

## 3. Discussion

Here, we present analysis of transcriptional repression of homologous type II toxin-antitoxin systems, *yefM-yoeB* and *axe-txe*. In our previously published studies, we have shown that these cassettes are negatively autoregulated at the transcriptional level, similarly to the vast majority of other known type II TA modules. Both antitoxins, Axe and YefM, play a role of the major repressor, while the toxins, Txe and YoeB, function as co-repressors [37,41]. However, in spite of the high sequence similarity of the main genetic promoter/operator elements, transcriptional activity and repression level of the major promoters of these TA cassettes, *p_yy_* and *p_at_*, was substantially different. Thus, unlike the *p_yy_* promoter of the *yefM-yoeB* system which is weaker and fully repressed by YefM-YoeB, the *p_at_* promoter of the *axe-txe* module turned out to be extremely strong and not efficiently inhibited by Axe-Txe [37,41].

In the report presented here we first established experimentally that the nucleotide sequence of the −35 box is responsible for the differences in the basal strength of *p_yy_* and *p_at_* promoters. This is in agreement with the data showing that in the consensus sequence for −35 motif the first three bases are the most conserved among promoters (5′-**TTG**ACA-3′) and that the more similar −10 and −35 boxes are to the consensus the stronger the promoter [5]. In case of *p_at_* all three nucleotides are the same as in the consensus sequence (5′-**TTG**ctt-3′), while for *p_yy_* the second T is replaced by A (5′-**TaG**ttA-3′) which explains a weaker activity of the latter. We have also tested the effect of transcriptional start site nucleotide, but changing A to G and *vice versa* did not exert any substantial effects on the promoter activity. This also confirms previously published data showing that both purine bases are more or less equally effective in transcription initiation [5].

Previously, we have also shown that despite the fact the proteins of *yefM-yoeB* and *axe-txe* modules appear to share high structure similarity, their cognate partners do not cross-interact [44]. Here, we revealed that the binding specificity of antitoxins and TA complexes to their own and homologous operator regions occurs on a similar level. It should not be surprising as both operator elements exhibit high resemblance in the major repressor binding sites, which are represented by two pairs of inverted repeats with identical core sequences (5′-TGTACA-3′), separated by the same distance of 12 bp from center to center. However, our previous results indicated a significant discrepancy between the repression level mediated by YefM-YoeB at the *p_yy_* promoter and by Axe-Txe at the *p_at_* promoter. While *p_yy_* inhibition was reaching almost the background level, *p_at_* repression was not efficient and still a substantial promoter activity could be detected. After careful analysis of both promoter/operator regions, we noticed that although both repressor binding palindromes were mostly identical in sequence, they were located differently in relation to the main promoter elements. This prompted us to suspect that maybe different steps in the transcription initiation process are perturbated in each case.

We decided to perform most of experiments only with YefM-YoeB proteins for several reasons. The presence of additional promoter (*p_axe_*) located within the *axe* antitoxin gene, which drives additional expression of the Txe toxin, makes it impossible to clone the *axe-txe* operon under a heterologous inducible promoter, like *p_BAD_* used in these studies [41]. This also makes it difficult to purify the Axe-Txe proteins, as TA proteins are usually overexpressed and purified as a complex. In our protein lysates used for the EMSA assays, the *axe-txe* genes were cloned together with the *p_at_* promoter downstream of the T7 promoter. It was the only way we found to overexpress Axe-Txe, however, the stoichiometry of both proteins does not have to be optimal and can differ from naturally occurring in the wild type system. Apart from that, this fusion does not contain a His-tag which would enable to purify the Axe-Txe complex as for an unknown reason a His-tagged version of Txe did not allow for overexpression of the complex. Nevertheless, EMSA assays with lysates obtained by overexpression of proteins of both systems clearly indicate that proteins of the *axe-txe* and *yefM-yoeB* systems are able to bind to their cognate and non-cognate operator regions with similar efficiency. The *p_at_* activity in the presence of Axe and Axe-Txe was also previously measured in *in cis lux*-transcriptional fusions [41] and these data are also analogous to those obtained in this manuscript with *in trans* fusions and YefM-YoeB proteins. In our opinion, these data allow us to assume that experiments with proteins of only one of these systems are sufficient to produce relevant answers about mechanism of transcriptional repression of both tested toxin-antitoxin cassettes.

In the literature, multiple examples of diversity observed in the organization of bacterial promoter-operator sites can be found. This suggests that prediction of a precise mechanism of transcription inhibition should be based not only on the operator sequence but rather on relative arrangements and topology of RNA polymerase and repressor on the DNA double helix. Varying binding site distribution for one repressor is quite common, however, cases where variations in the operator positioning result in different repression mechanism are not frequently reported. There are many examples of repressors that hinder RNAP binding to promoters since their operator site/s precisely overlap promoter elements recognized by the sigma subunit of RNAP (−10 or −35 boxes or transcription start site), including the well-studied Fur repressor of *E. coli* or bacteriophage λ CI repressor at the *p_R_* promoter [49,50]. Whereas only a few repressors are known that bind the operator sequence located within or in close proximity of the promoter simultaneously with RNAP and freeze later steps in transcription initiation. For example, phage Φ29 p4 protein, GalR repressor and KorB protein were shown to inhibit transcription by binding upstream of −35 box and prevent promoter open complex formation or promoter clearance by interacting with αCTD subunit of RNA polymerase [51,52,53]. On the other hand, operator sites for *Bacillus subtilis* Spo0A protein lie downstream relative to the transcription start site and RNAP is unable to induce strand separation for open complex formation [54].

The primary binding site for YefM-YoeB complex at the *p_yy_* promoter, the L repeat, overlaps the −10 promoter box, while the S repeat encompasses the transcription start site. That kind of placement of the operator sequence suggests creation of a steric hindrance for RNA polymerase binding. Such mechanism of transcription repression has been confirmed by our EMSA and DNaseI footprinting experiments where the bound repressor either prevented RNAP from binding at the same time or did not allow RNAP to effectively displace it. On the other hand, in our studies, transcription inhibition mediated by the YefM-YoeB or Axe-Txe complexes at *p_at_* was not as efficient as at the *p_yy_* promoter, despite identical binding core sequences in both operators. We propose that the reason for that is different location of both operator sites relative to the promoter recognition elements resulting in different repression mechanisms. At *p_at_* the major repressor binding site, the L repeat, lies within the linker between −10 and −35 promoter boxes, while the S repeat partly overlaps the −10 hexamer. Such operator sequence location potentially enables simultaneous RNAP and repressor binding and transcription inhibition occurs at a later step. Binding site/s located within promoter spacer have been already identified for few repressors. For example, Arc protein of *Salmonella typhimurium* phage 22, MerR repressor and nucleoid-associated Fis protein have been demonstrated to slow the rate at which RNAP forms open complex or to inhibit promoter opening by trapping RNAP at a promoter [55,56,57].

To our knowledge, in all examples described to date where the repressor protein binds to the promoter spacer sequence, it influences the open complex formation step in the transcription initiation process. This is not surprising, since it has been shown that for effective promoter opening physical deformations causing conformational changes within the linker DNA occur in parallel with melting of the promoter sequence extending from the −10 box [58]. Thus, any protein factor bound at the linker in some way potentially affects the open complex formation step. Based on the results of our EMSA and DNaseI footprinting assays we propose that the binding of the Axe-Txe/YefM-YoeB repressor complex to its operator sites placed within the spacer of the *p_at_* promoter enables simultaneous binding of the RNA polymerase and probably affects the open complex formation step. Repression at this step of transcription initiation seems to be less efficient than complete blocking of RNAP binding, like in the *p_yy_* example. Transcriptional autorepression is a characteristic feature of a vast majority of type II toxin-antitoxin systems, however to date a precise stage in the multistep transcription initiation process which is affected by repressor binding was not investigated in any of the known examples.

Another element which we found to be different in the *yefM-yoeB* and *axe-txe* regulation is the presence of an additional active promoter located 40 bp upstream of the major *p_at_* promoter, which we called *p_at2_*. Its presence was discovered by in vitro transcription experiments where we noticed a band above the main transcript. To confirm this promoter’s activity in vivo*,* we have used transcriptional fusions with the *lux* reporter gene, which were devoid of the sequence fragment upstream of the *p_at_* promoter, including this promoter region. Transcription activity measured just for the main *p_at_* promoter appeared to be lower by around 28% in comparison with the sample where the two promoters were present. Its presence and activity was also confirmed in in vitro transcription assays. These experiments also indicate that *p_at2_* activity masks the repression level mediated by the TA complex at the *p_at_* promoter, since it does not seem to be regulated by this protein complex. Results presented in Figure 10B,C show that the more *p_at_* promoter is repressed by the YefM-YoeB complex, the higher transcript level initiating from the *p_at2_* promoter. We assume that this results from more RNAP molecules becoming available for binding on this DNA region and is not due to a direct activation by the TA complex. Computational analysis of *E. coli* and other bacterial species regulatory regions revealed that they contain clusters of promoter-like signals, in contrast to the coding regions. In other words, functional promoters occur mostly within regions with high density of overlapping putative promoters [14]. With great specificity, RNA polymerase identifies precise promoter sites in the middle of this jungle of plausible attractive sites. The role of such multiple overlapping promoter-like sequences may be to channel RNAP to the active promoter region. Moreover, it has been estimated that 25% of transcription units have more than one functional promoter [15]. One can imagine that such multiple promoter signals may play diverse roles depending on their relative distance, strength, orientation and presence of binding sites for additional regulatory factors. There are several examples of regions reported to have more than one functional promoter that transcribe the same downstream gene. Such tandem promoters can play regulatory roles, which is negative if those sites have a competitive relationship, or positive if they have additive effect on overall transcription of the downstream gene. Moreover, adjacent promoters may be regulated independently by the action of different activators or repressors and thereby allow to precisely regulate expression of a gene in response to different environmental stimuli, i.e., they become active under particular conditions.

There are only single examples of known class II TA cassettes whose transcription is directed by two adjacently positioned promoters. The *E. coli mazEFG* module is preceded by two promoters located 13 bp apart, *P2* and *P3*, whose relative strengths differ by approximately ten-fold [59,60]. Transcription from both promoters is repressed by the MazE-MazF complex. The action of these two promoters was shown to be additive [60]. The *yefM-yoeB* operon of *Streptococcus pneumoniae* provides another example of transcriptional control mediated by a pair of promoters located upstream of these TA genes. Both promoters are located 30 nt apart from each other. In this case, the expression from one of them, *p_yefM1_* is constitutive while the other, stronger promoter, situated more downstream, *p_yefM2_*, is negatively autoregulated by YefM-YoeB complex. *P_yefM1_* was found to be 15-fold weaker than *p_yefM2_*, and its presence was shown to decrease the overall promoter activity in *yefM-yoeB* operon [39]. The *hicAB* locus of *E. coli* was also shown to be transcribed from two promoters [61]. It has been established that an upstream promoter, *P1*, generates a *hicAB* transcript that produces both HicA and HicB. On the other hand, the downstream *P2* promoter is autorepressed solely by HicB antitoxin and produces only HicB but not HicA toxin. These results indicate that the *P1* and *P2* generated transcripts are translated at highly different rates [61]. Transcription start sites of both promoters lay 54 bp apart from each other.

As we have not found any obvious regulatory elements upstream of the *p_at2_* promoter we cannot speculate about its regulation. However, it appears that the regulation of *axe-txe* expression in terms of the regulatory elements located upstream of the *axe* gene resembles the situation found in streptococcal *yefM-yoeBSpn*, where the strong main *p_at2_* promoter is precedent by a weaker, constitutive promoter *p_at1_* [39]. This may also be the case for *p_at2_*.

During formation of a loose, unstable initial closed complex (RPc) RNAP protects the promoter DNA from position −55 to −5 (around 50 nt). When RNA polymerase forms transcriptionally active complex (open complex) it covers the promoter sequence extending from about −50 to +20 (around 70 nt) in respect to the transcription start site. Since the distance between the *p_at_* and *p_at2_* promoters is 40 bp it suggests that transcription from both promoters cannot occur simultaneously and is rather subjected to some kind of competition. Moreover, it is likely that both promoters are positioned on the same face of the DNA double helix since the distance between them is close to four full helical turns. RNA polymerase molecules may bind simultaneously to both promoter sites however at *p_at2_* only closed complexes could be formed, while at *p_at_* one αCTD of RNAP would be possibly displaced. Thus, it seems possible that each promoter may influence the other’s binding by RNAP and thus modulate the overall promoter activity of the *axe-txe* transcription unit. In the future it would be worth elucidating the interplay between them by mutational analyses where each of these two adjacent promoters would be inactivated and tested under repressive and non-repressive conditions.

**Figure 11:**
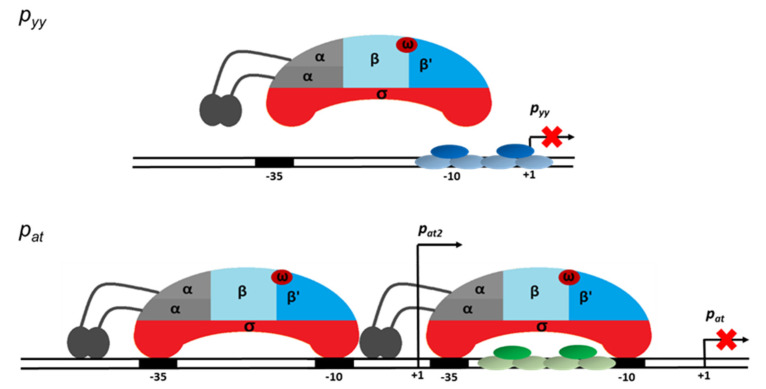
Model of transcription repression mediated at the *p_yy_* (upper panel) and *p_at_* (lower panel) promoter regions. YefM-YoeB and Axe-Txe repressor trimer complexes bound to their operator DNAs are depicted in blue and green ovals, respectively. The red crosses indicate block of transcription process. Detailed explanation of the proposed mechanisms of transcription repression of both investigated promoters is presented in the text.

Based on the results presented here we propose a model of transcriptional regulation of two highly homologous TA systems, YefM-YoeB and Axe-Txe, in which nucleotide sequences of core repressor binding sites are identical, however, they are positioned differently in relation to the major promoter anchors for the σ^70^ subunit of RNA polymerase. At *p_yy_*_,_ steric hindrance occurs, repressor binding is strong, and RNAP cannot reach the crucial promoter elements and thus repression is efficient. In contrast, at *p_at_*, the repressor binding site only partially overlaps with the RNAP binding elements, so both protein complexes can bind DNA at the same time, and thus repression occurs at further stages of transcription initiation and this repression mechanism is not as effective. Moreover, a second promoter located upstream of *p_at_*, called *p_at2_*, contributes to gene expression of the *axe-txe* module and its transcription does not seem to be directly regulated by the TA complex bound to the main promoter (Figure 11). This explains the need of the *axe-txe* system for additional regulatory elements which would provide a proper balance in the production of the toxin and antitoxin. Thus, the presence of additional internal promoters within the *axe* and *txe* genes, the latter in the reverse orientation along with a terminator-like sequence which is probably responsible for the decreased stability of the transcript, secure appropriate functioning of the *axe-txe* module [41].

## 4. Materials and Methods

### 4.1. Strains, Plasmids and Oligonucleotides

*E. coli* DH5α was used for plasmid construction and purification. Rosetta (DE3) was used for crude extract preparation with Axe, Axe-Txe, YefM and YefM-YoeB overproduction from pET22axe and pET22at_axe-txe, pET22yefM and pET22yefM-yoeB, respectively. BL21(DE3) was used for YefM-YoeB6His overproduction and purification. Strain SC301467, a derivative of MG1655 devoid of *mazF*, *chpB*, *relBE*, *dinJ-yafQ* and *yefM-yoeB* [46] was used for luminescence assays with appropriate derivatives of the pBBRlux-amp plasmid [37]. *E. coli* cells were grown in Luria-Bertani (LB) medium at 37 °C with shaking. Ampicillin and chloramphenicol were added to final concentrations of 100 µg/mL and 34 µg/mL, respectively, when required. *Enterococcus faecalis* strain OG1RF was used for β-galactosidase assays with derivatives of the pTCVlac vector [62]. For these experiments enterococcal cultures were grown in the BBL Trypticase Soy Broth with kanamycin added to final concentration of 500 µg/mL at 37 °C without shaking. Plasmids and oligonucleotides used are listed in Tables S1 and S2, respectively. All oligonucleotides were ordered from Sigma Aldrich/Merck (Darmstadt, Germany) and all restriction enzymes were purchased from Fermentas/ThermoFisher Scientific (Waltham, MA, USA). All plasmid constructs were verified by sequencing (Macrogen Europe, Amsterdam, The Netherlands).

### 4.2. Crude Extract Preparation

Bacteria were grown at 37 °C in 10 mL of LB medium with appropriate antibiotic until OD_600_~0.5. Expression of *axe* (pET22axe), *axe-txe* (pET22at_axe-txe), *yefM* (pET22yefM) or *yefM-yoeB* (pET22yefM-yoeB) was induced with 1 mM IPTG and incubation continued for 3 h. Cells were harvested at 1600× *g* for 10 min. The pellet was resuspended in 1 mL of buffer containing 20 mM Tris-HCl pH 7.5 and 50 mM NaCl. The cells were sonicated and then centrifuged for 30 min at 15,500 × *g* at 4 °C. Supernatant was dialyzed against the same buffer containing 10% glycerol. Glycerol was added to the samples up to 50%, which were then aliquoted and stored at −20 °C.

### 4.3. YefM-YoeB Protein Complex Purification

YefM-YoeB complex was overproduced in *E. coli* BL21(DE3) and purified by Ni^2+^ affinity chromatography essentially according to the Novagen technical manual. 150 mL of culture harboring the expression plasmid pETyefM-yoeB was grown at 37 °C until OD_600_ ≈ 0.8, expression was induced with 1 mM IPTG and growth continued for 3 h. Cells were harvested at 4000 rpm at 4 °C for 10 min. The pellet was resuspended in 4 mL of binding buffer (10 mM imidazole, 20 mM Tris-HCl pH 8.0, 500 mM NaCl) with lysozyme (0.1 mg/mL). Next, the cells were sonicated and then centrifuged for 1 h at 15,000 rpm at 4 °C. The supernatant was applied to a column containing 2 mL of His-tag resin (Roth) and equilibrated with the binding buffer. Binding of the fusion protein to the resin was allowed to continue for 1 h at 4 °C after which the column was washed with 15 mL of wash buffer (50 mM imidazole, 20 mM Tris-HCl pH 8.0, 500 mM NaCl). Elution was performed with 5 mL of elution buffer (250 mM imidazole, 20 mM Tris-HCl pH 8.0, 500 mM NaCl) and 1 mL fractions were collected. Two fractions containing the YefM-YoeB complex were combined and dialyzed against 1 L of storage buffer (50 mM Tris-HCl pH 8.5, 150 mM NaCl, 10% glycerol) overnight, and then for 2 h against fresh storage buffer. Glycerol was added to the samples up to 50%, which were then aliquoted and stored at −20 °C.

### 4.4. RNA Polymerase Holoenzyme Purification

Native RNA polymerase from *E. coli* was purified as described [63] with modifications as in [64].

### 4.5. Promoter Fusion Studies and Bioluminescence Assays in Escherichia coli Cells

Strain SC301467 harboring derivatives of pBBRlux-amp with the *lux* operon under transcriptional control of the wild-type and mutated *p_yy_* or *p_at_* promoter fragments were used. PCR fragments were cloned into pBBRlux-amp between *Spe*I-*BamH*I restriction sites upstream of the promoterless *luxCDABE* to yield proper transcriptional fusions. Where indicated, the strains with pBBRlux fusions were additionally co-transformed with pBAD33 plasmids [65] encoding the *yefM* or *yefM-yoeB* genes under control of an arabinose-inducible promoter (pBADyefM and pBADyefMyoeB). Overnight cultures carrying recombinant plasmids were diluted (1:100) into fresh LB medium and grown until OD_600_~0.5. Where necessary, synthesis of antitoxin or toxin-antitoxin was induced by addition of 0.2% L-arabinose at time of culture dilution. Then, luminescence of 200 µL of cell cultures was measured in a luminometer (Berthold Technologies, Junior, Bad Wildbad, Germany). Results, obtained in relative light units (RLU), were divided by the optical density (OD_600_) of the cultures.

### 4.6. Electrophoretic Mobility Shift Assays (EMSA)

Cy5-labeled double-stranded PCR fragments that included the *p_at_* (primers 156 and 157) and *p_yy_* (primers 154 and 155) promoter regions were used in EMSA studies. Reactions containing 10 nM of cy5-labeled DNA and bacterial crude extract or purified YefM-YoeB6His complex were assembled in binding buffer (10 mM Tris-HCl pH 7.5, 100 mM NaCl, 1 mM DTT, 5 mM MgCl_2_, 1 µg of poly(dIdC), 2.5% glycerol) in final volumes of 20 µL and incubated for 20 min at RT. Then samples were electrophoresed on 6% native polyacrylamide gels in 0.5× TBE buffer for 120 min at 100 V at RT. Detection of the cy5–labeled DNA was performed using Typhoon scanner. Competitive EMSA with YefM-YeoB6His complex and *E. coli* RNA polymerase holoenzyme were performed on cy-5 one strand labelled DNA fragments (*p_at_*—primers 86 and 157, *p_yy_−*35mut—primers 84 and 155) in the reaction mixture and conditions described above, however samples were run on 4% native polyacrylamide gels in 0.5× TBE buffer for 70 min at 100 V at RT.

### 4.7. In vitro Transcription Analysis

Transcription activity of *p_at_* and *p_yy_* promoters was analyzed in multiround in vitro transcription assays performed with circular plasmid DNAs (derivatives of the pTE103 vector [66]) as indicated in the figures. Reactions were done at 37 °C in total volumes of 20 µL and contained 40 mM Tris-HCl pH 8.0, 200 mM KCl, 10 mM MgCl_2_, 10 mM DTT, 10 µg BSA, 5 nM DNA. Moreover, *E. coli* σ^70^ RNA polymerase holoenzyme (RNAP) (25 nM) and YefM-YoeB6His complex were added in the indicated order and samples were incubated for 10 min. The reactions were started by addition of ribonucleotides, final concentrations: 0.15 mM of GTP, ATP and CTP, 0.015 mM of UTP and 0.8 µCi α^32^P-UTP, and were run for 15 min. Then, 20 µL of stop solution (95% formamide, 0.5 M EDTA, 0.05% bromophenol blue) was added and samples were denatured for 5 min at 95 °C prior to loading 20 µL on a denaturing 7 M urea, 6% polyacrylamide gel, and quantified by phosphor-imaging with a Typhoon 9200 imaging system (GE Healthcare, Bio-Sciences AB, Sweden).

### 4.8. DNaseI Footprinting Assays

For these assays, protein-DNA binding reactions were performed in 20 µL in the following buffer: 50 mM Tris-Cl (pH 8.0), 10 mM MgCl_2_, 100 mM NaCl, 1 mM β-mercaptoethanol. PCR fragments with 5′ cy5 label and containing the *p_yy_* (primers 154 and 181) or *p_at_* (primers 156 and 181) promoters were used as templates (20 nM final concentration). To stabilize the RNAP-DNA open complexes, ATP and UTP (for *p_at_*) and GTP and UTP (for *p_yy_*) were added to 125 µM. RNAP was used at 0, 50, 100, 150, 200 nM, and YefM-YoeB6His was used at 1 µM. When RNAP or YefM-YoeB6His were assayed alone, the samples were incubated at 37 °C for 10 min, followed by an addition of DNase I (0.075 units/20 µL sample; Eurx, Gdańsk, Poland) and further incubation at 37 °C for 2.5 min. In reactions where RNAP and YefM-YoeB6His were assayed together, one or the other was pre-incubated at 37 °C with DNA in the reaction buffer for 10 min, followed by the addition of the other protein and further incubation for another 10 min. Reactions were terminated by addition of EDTA to 25 mM and concentrated by vacuum evaporation. Next, samples were resuspended in 14 µL of loading solution (80% formamide, 6 M urea, 10 mM NaOH), denatured at 100 °C for 2 min, and 7 µL were loaded on 7 M urea, 8% acrylamide gels along with sequencing reactions (5′Cy-5 labelled primers were employed, 156 for *p_at_* and 154 for *p_yy_*, with the DNA Cycle Sequencing Kit, Jena Bioscience, Jena, Germany). Typhoon image system (GE Healthcare) was used for scanning and documentation.

### 4.9. Promoter Fusion Studies and β-Galactosidase Assays in Enterococcal cells

Electrocompetent cells of OG1RF strain were electroporated with derivatives of pTCVlac vector bearing the *lacZ* gene under transcriptional control of the *p_at_* or *p_at2_p_at_* promoter fragments. PCR fragments were cloned into pTCVlac [62] between *EcoR*I and *BamH*I restriction sites upstream of promoterless *lacZ* gene. Overnight cultures carrying recombinant plasmids were diluted (1:50) into fresh BBL Trypticase Soy medium and grown until OD_600_~0.5. Then β-galactosidase activity assay was performed with cells permeabilized with chloroform and SDS as described by Miller [67]. We found this method for cell permeabilization to be more efficient than the one with toluene and ethanol, which was described previously for enterococcal cultures [62].

### 4.10. Data Analysis

Promoter searches were performed using PromScan bioinformatic program (http://molbiol-tools.ca/promscan/).

## 5. Conclusions

In most cases genes are not simply turned on or off, but instead their expression is precisely fine-tuned to suit the demands of the cell in response to changing environmental conditions, as well as to keep intracellular balance. How genes are modulated so precisely is still not well understood, but it appears that mechanisms driving this regulation are more complex and multilayered than previously expected. This is because many of these regulatory mechanisms/elements produce a subtle effect/change that easily can be overlooked or unnoticed under standard experimental conditions/procedures. It is obvious that in many cases only the major—i.e., giving the strongest effect—elements are being studied and described. Due to the engagement of type II TAs in virulence, antibiotic tolerance and biofilm formation, it is crucial to understand how these genes are regulated at a molecular level. Our work substantially adds to these studies.

## Supplementary Materials

The following are available online at https://www.mdpi.com/1422-0067/21/23/9062/s1.
