# Supplementary material for: Insights into Transcriptional Repression of the Homologous Toxin-Antitoxin Cassettes yefM-yoeB and axe-txe
Source: Int J Mol Sci. 2020 Nov 28;21(23):9062. doi: 10.3390/ijms21239062 (PMC7730913; doi:10.3390/ijms21239062)
Supplement: Supplementary file 1 [file ijms-21-09062-s001.pdf]

Insights into transcriptional repression of the homologous toxin-antitoxin cassettes *yefM-yoeB* and *axe-txe*

Barbara Kędzierska [1,*], Katarzyna Potrykus [1], Agnieszka Szalewska-Pałasz [1] and Beata Wodzikowska [1]

YefM6His – 89 aa - 10,13 kDa

YefM – 83 aa – 9,31 kDa

YoeB6His – 90 aa – 11,04 kDa

Axe6His – 95 aa – 11,2 kDa

Axe – 89 aa – 10,38 kDa

Txe – 85 aa – 10,21 kDa

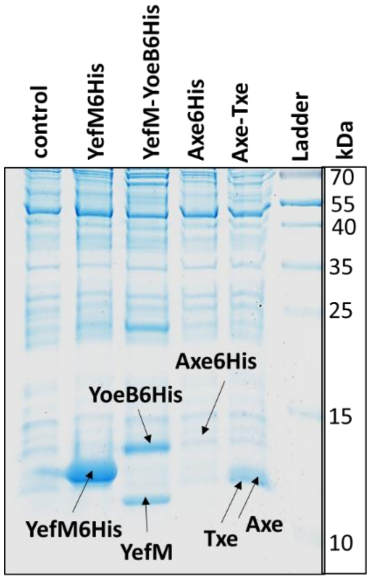

Figure S1. 15% SDS-PAGE shows the indicated proteins' overproduction in lysates of BL21(DE3) cells transformed with appropriate pET22(b) derivatives. PageRuler Prestained Protein Ladder (Thermo Scientific) was loaded in the last lane. Appropriate molecular weights of the protein marker are indicated in kDa.

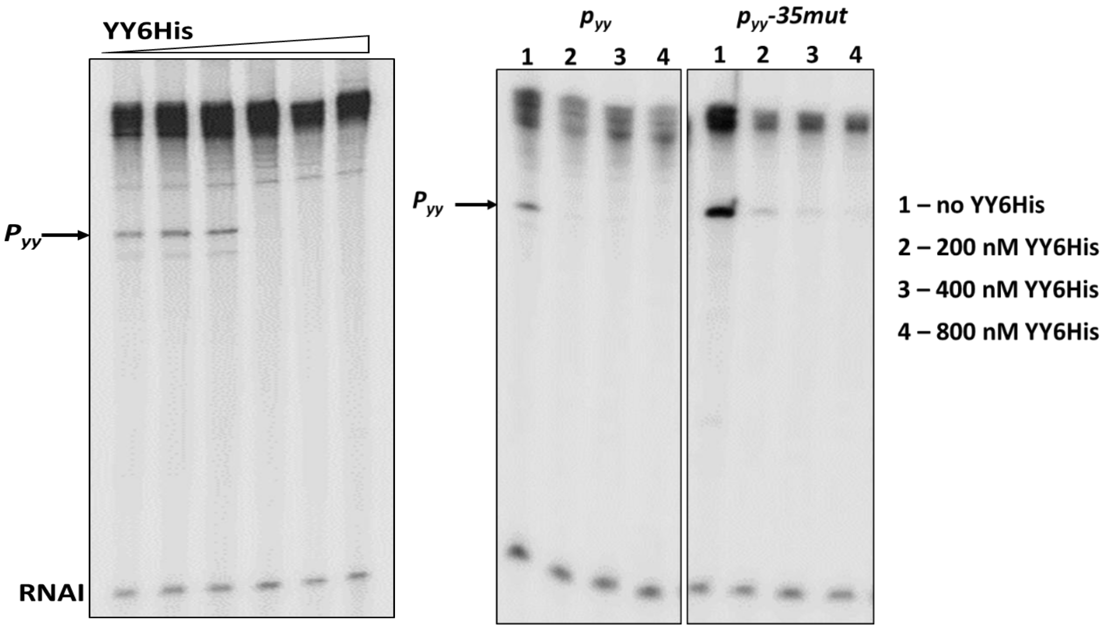

Figure S2. Transcriptional repression of p_{yy} by the YefM-YoeB complex *in vitro*. Multiround *in vitro* transcription reactions were performed on 5 nM pTEpyy DNA template, using 25 nM *E. coli* σ^{70} RNA polymerase holoenzyme and increasing concentrations of YefM-YoeB6His complex (0, 20, 100, 200, 1000, 2000 nM) – left panel. *In vitro* transcription reactions on pTEpyy and pTEyy-35mut DNA templates with increasing concentrations of YefM-YoeB6His complex (0, 200, 400, 800 nM) – right panels. Reactions were carried out and analyzed as outlined in Materials and Methods.

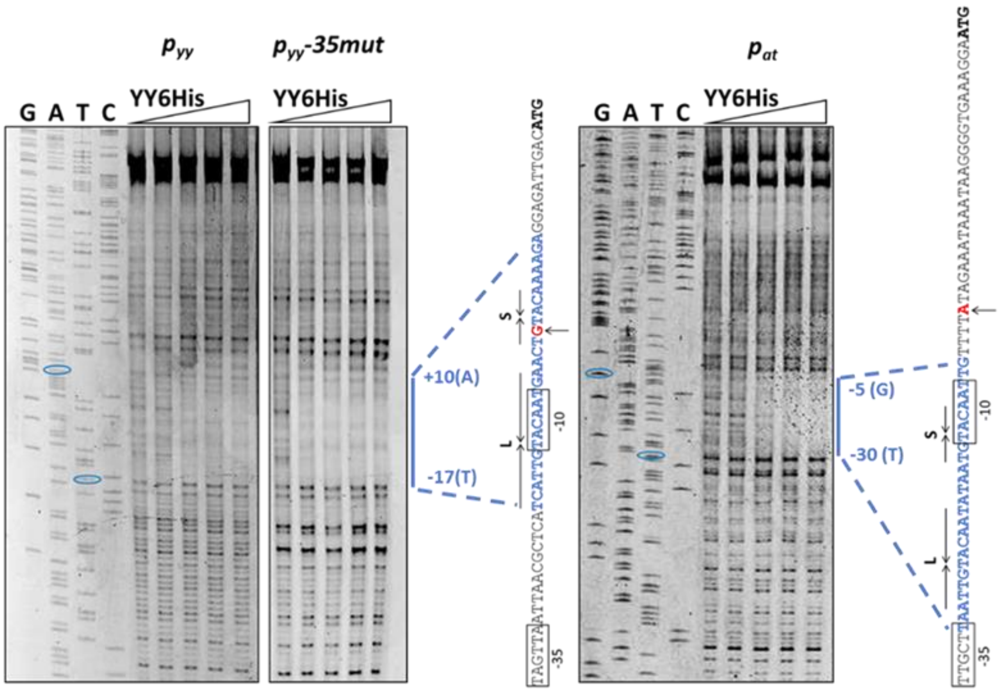

Figure S3. DNaseI footprinting of the YefM-YoeB complex on p_{yy} wt, p_{yy}-35mut and p_{at} promoter-operator regions. Footprinting reactions were performed as outlined in Materials and Methods using increasing concentrations of YefM-YoeB6His (0, 0.25, 0.5, 1, 2 μM) bound to 20 nM cy5-labeled appropriate DNA fragment for 10 min at 37⁰C. The samples were then treated with DNaseI and reactions were run on 8% denaturing acrylamide gels along with sequencing reactions (GATC). The relative positions of regions on the upper strand that is protected from DNaseI digestion are illustrated on the right side of the gels.

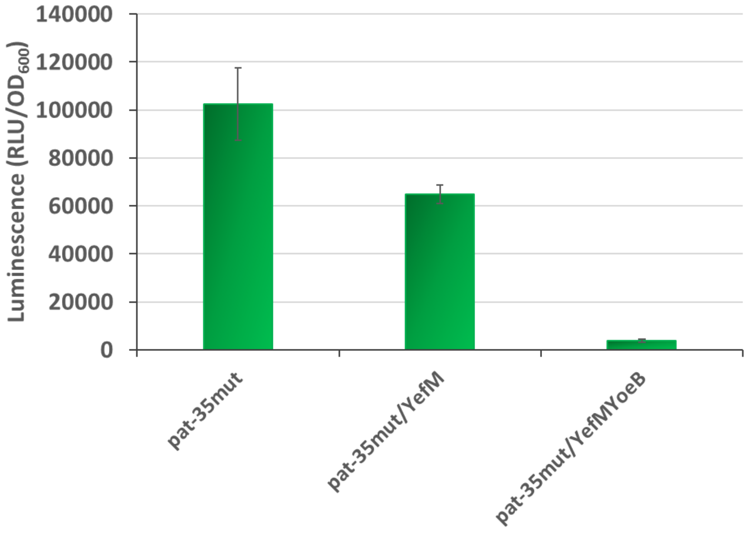

Figure S4. Transcription repression of p_{at}-35mut mediated by YefM and YefM-YoeB *in vivo.* Transcriptional *lux* gene fusions of p_{at}-35mut promoter were made in pBBRlux-amp vector and were introduced into the SC301467 strain together with pBAD33 vector control (for promoter unrepressed conditions – first bars) and pBAD33 derivatives producing YefM or YefM-YoeB complex. Assays were conducted after induction with 0.2% L-arabinose. Luminescence was measured in RLU (relative luminescence units) when cell cultures reached OD_{600}~0.5. The basal activity of pBBRlux-amp vector control was ~100 units. These results are average of three independent experiments.

Table S1. Plasmids used in this work.

Plasmid	Description	Source
pBBRlux-amp	Vector for generating transcriptional fusion to *lux*, *bla* gene was cloned into *EcoR*I site within the *cat* gene, Amp^R	[41]
pBBRlux_pat	p_{at} promoter-operator region, digested with *Spe*I-*BamH*I and cloned between equivalent sites in pBBRlux-amp	[41]
pBBRlux_pyy	p_{yy} promoter-operator region, amplified with primers 84 and 85, digested with *Spe*I-*BamH*I and cloned between equivalent sites in pBBRlux-amp	This work
pBBRlux_pat+1mut	The same as pBBRlux_pat, but p_{at} promoter fragment was amplified with primers 76 and 38 on a pTEpat+1mut template and cloned between *Spe*I and *BamH*I sites	This work
pBBRlux_pyy+1mut	The same as pBBRlux_pyy, but p_{yy} promoter fragment was amplified with primers 84 and 85 on a pTE*pyy*+1mut template and cloned between *Spe*I and *BamH*I sites	This work
pBBRlux_pat-35mut	The same as pBBRlux_pat, but p_{at} promoter fragment was amplified with primers 76 and 38 on a pTEpat-35mut template and cloned between *Spe*I and *BamH*I sites	This work
pBBRlux_pyy-35mut	The same as pBBRlux_pyy, but p_{yy} promoter fragment was amplified with primers 84 and 85 on a pTEpyy-35mut template and cloned between *Spe*I and *BamH*I sites	This work
pBAD33	Arabinose-inducible expression vector, Cm^R	[65]
pBADyefM	*yefM* gene cloned into pBAD33 between *Hind*III and *Xho*I restriction sites, under control of L-arabinose inducible promoter	[37]
pBADyefMyoeB	*yefM-yoeB* cassette cloned into pBAD33 between *Hind*III and *Xho*I restriction sites, under control of L-arabinose inducible promoter	[37]
pTE103	Vector for generating transcription templates, contains the multicloning site placed upstream from a bacteriophage T7 transcriptional terminator, Amp^R	[66]
pTEpat	p_{at} promoter fragment (bearing p_{at2} and p_{at1} sequences) amplified with primers 37 and 38 and cloned between *EcoR*I and *BamH*I restriction sites of pTE103	This work
pTEpyy	p_{yy} promoter fragment amplified with primers 72 and 85 and cloned between *EcoR*I and *BamH*I restriction sites of pTE103	This work
pTEpat+1mut	The same as pTEpat, but transcription start site (+1) of p_{at} was changed from A to G with primers 169 and 170	This work
pTEpyy+1mut	The same as pTEpyy, but transcription start site (+1) of p_{yy} was changed from G to A with primers 167 and 168	This work
pTEpat-35mut	The same as pTEpat, but -35 box of p_{at} was changed from TTGCTT to TAGTTA with primers 165 and 166	This work
pTEpyy-35mut	The same as pTEpyy, but -35 box of p_{yy} was changed from TAGTTA to TTGCTT with primers 163 and 164	This work
pET22b	IPTG-inducible expression vector allowing fusion of C-terminal 6His-tag to the target protein, Amp^R	Novagen

pETyefM	*yefM* cloned between *Nde*I and *Xho*I restriction sites in pET22b, resulting YefM with C-terminal 6His-tag	[37]
pETyefM-yoeB	*yefM-yoeB* cloned between *Nde*I and *Xho*I restriction sites in pET22b, resulting YefM-YoeB complex with C-terminal 6His-tag on YoeB	[37]
pETaxe	*axe* cloned between *Nde*I and *Xho*I restriction sites in pET22b, resulting Axe with C-terminal 6His-tag	[41]
pETpat_axe-txe	*axe-txe* (with p_{at} promoter) cloned between *Nde*I and *Xho*I restriction sites in pET22b, resulting Axe-Txe complex (without 6His-tag)	[41]
pTEpat1	Promoter fragment bearing only p_{at1} promoter sequence (without p_{at2} promoter sequence) was amplified with primers 93 and 38 and cloned between *EcoR*I and *BamH*I restriction sites of pTE103	This work
pTEpat2mut-10pat1	The same as pTEpat, but -10 box of p_{at2} was changed from TAGAAT to CGGAAT with primers 201 and 202	This work
pTCVlac	Broad-host-range low copy number shuttle vector for construction of transcriptional fusion with promoterless *lacZ* gene (bearing Gram+ ribosome binding site) to study regulatory elements in gram positive bacteria, Kan^R, Erm^R	[62]
pTCVlac_pat1	Promoter fragment bearing only p_{at1} promoter sequence (without p_{at2} promoter sequence) was amplified with primers 93 and 38 and cloned between *EcoR*I and *BamH*I restriction sites of pTCVlac	This work
pTCVlac_pat2pat1	Fragment bearing $p_{at2}p_{at1}$ promoters sequence was amplified with primers 37 and 38 and cloned between *EcoR*I and *BamH*I restriction sites of pTCVlac	This work

Table S2. Oligonucleotides used in this study. Restriction enzyme sites and mutated positions are underlined.

Name	Sequence (5′-3′)	Description
**84**	GAGTACTAGTGGGGAAAGGAGGGGG	Forward primer for amplification of p_{yy} promoter fragment to pBBRlux-amp, *Spe*I restriction site; forward primer for one strand labelled EMSA p_{yy} and p_{yy}-35mut promoter fragment
**85**	CATTCGGATCCTGACGCGCTTCGCTG	Revers primer for amplification of p_{yy} promoter fragment to pBBRlux-amp and pTE103, *BamH*I restriction site
**38**	CCAAGGATCCGAATAAGCTACTGCTTCC	Revers primer for amplification of p_{at} promoter fragment to pBBRlux-amp and pTE103 and pTCVlac, *BamH*I restriction site
**76**	GAGTACTAGTGAAAAAGCAGGATTTGAGG	Forward primer for amplification of p_{at} promoter fragment to pBBRlux-amp, *Spe*I restriction site
**37**	GAGTGAATTCGAAAAAGCAGGATTTGAGG	Forward primer for amplification of p_{at} promoter fragment to pTE103 and pTCVlac, *EcoR*I restriction site
**72**	CGCGGGAATTCGGGGAAAGGAGGGGG	Forward primer for amplification of p_{yy} promoter fragment to pTE103, *EcoR*I restriction site
**169**	CAATATAATGTACAATTGTTTTGTAGAAATAAATAAGGG	Forward primer for +1 site mutagenesis of p_{at} promoter
**170**	CCCTTATTTATTTCTACAAAACAATTGTACATTATATTG	Reverse primer for +1 site mutagenesis of p_{at} promoter
**167**	CATTGTACAATGAACTATACAAAAGAGGAGATTG	Forward primer for +1 site mutagenesis of p_{yy} promoter
**168**	CAATCTCCTCTTTTGTATAGTTCATTGTACAATG	Reverse primer for +1 site mutagenesis of p_{yy} promoter
**165**	GTTAGAATATTAATCATTAGTTAAATTGTACAATATAATG	Forward primer for -35 box mutagenesis of p_{at} promoter
**166**	CATTATATTGTACAATTTAACTAATGATTAATATTCTAAC	Reverse primer for -35 box mutagenesis of p_{at} promoter
**163**	CTACAAACTAATTAATAAATTGCTTATTAACGCTCATCAT	Forward primer for -35 box mutagenesis of p_{yy} promoter
**164**	ATGATGAGCGTTAATAAGCAATTTATTAATTAGTTTGTAG	Reverse primer for -35 box mutagenesis of p_{yy} promoter
**154**	[cy5]AGCTGATGGCACCAAAAATAGCG	Forward primer for cy5 p_{yy} promoter fragment, for DNaseI footprinting and EMSA
**155**	[cy5]TTGTTGCCGACAAATTCTGACG	Reverse primer for cy5 p_{yy} promoter fragment for EMSA
**156**	[cy5]AGCAGGATTTGAGGTACCAGC	Forward primer for cy5 p_{at} promoter fragment, for DNaseI footprinting and EMSA
**157**	[cy5]TCATATAACTACGTAAATTTTGGCGG	Reverse primer for cy5 p_{at} promoter fragment for EMSA
**86**	TTTCCCAGTCACGACGTTG	Forward primer for one strand labelled competitive EMSA on p_{at} promoter fragment
**181**	ATTAAGGCGCGAGAGATATTCG	Reverse primer for generating p_{at} and p_{yy} fragments for DNaseI footprinting

**93**	GATC<u>GAATTC</u>ATCATTTGCTTAATTG	Forward primer for amplification of pat1 promoter fragment to clone to pTE103 and pTCVlac vectors, *Eco*RI restriction site
**201**	TAATTATAAGAAGT<u>CG</u>GAATATTAATC	Forward primer for mutagenesis of p_{at2} promoter -10 box
**202**	GATTAATATTC<u>CG</u>ACTTCTTATAATTA	Reverse primer for mutagenesis of p_{at2} promoter -10 box
